# Frequency controlled agglomeration of pt-nanoparticles in sonochemical synthesis

**DOI:** 10.1016/j.ultsonch.2022.105991

**Published:** 2022-03-31

**Authors:** Henrik E. Hansen, Frode Seland, Svein Sunde, Odne S. Burheim, Bruno G. Pollet

**Affiliations:** a Electrochemistry Group, Department of Materials Science and Engineering, Faculty of Natural Sciences, Norwegian University of Science and Technology (NTNU), NO-7491 Trondheim, Norway; b Hydrogen Energy and Sonochemistry Research Group, Department of Energy and Process Engineering, Faculty of Engineering, Norwegian University of Science and Technology (NTNU), NO-7491 Trondheim, Norway; c Green H_2_ Lab, Pollet Research Group, Hydrogen Research Institute (HRI), Université Du Québec à Trois-Rivières (UQTR), 3351 Boulevard des Forges, Trois-Rivières, Québec G9A 5H7, Canada

**Keywords:** Ultrasound, Sonochemistry, Electrocatalyst, Hydrogen, Platinum, Frequency

## Abstract

Optimizing the surface area of nanoparticles is key to achieving high catalytic activities for electrochemical energy conversion devices. In this work, the frequency range (200 kHz–500 kHz) for maximum sonochemical radical formation was investigated for the sonochemical synthesis of Pt-nanoparticles to assess whether an optimum frequency exists or if the entire range provides reproducible particle properties. Through physical and electrochemical characterization, it was found that the frequency dependent mechanical effects of ultrasound resulted in smaller, more open agglomerates at lower frequencies with agglomerate sizes of (238 ± 4) nm at 210 kHz compared to (274 ± 2) nm at 326 kHz, and electrochemical surface areas of (12.4 ± 0.9) m^2^g^−1^ at 210 kHz compared to (3.4 ± 0.5) m^2^g^−1^ at 326 kHz. However, the primary particle size (2.1 nm) and the catalytic activity towards hydrogen evolution, (19 ± 2) mV at 10mA cm^2^,remained unchanged over the entire frequency range. Highly reproducible Pt-nanoparticles are therefore easily attainable within a broad range of ultrasonic frequencies for the sonochemical synthesis route.

## 1 Introduction

The synthesis of nanomaterials with highly reproducible physical properties has been a challenge ever since researchers started to explore their unique capabilities [1], [2]. Most synthesis techniques are very sensitive towards changes in the experimental conditions and can yield nanoparticles with very different properties which might not be ideal for the given application it is intended for. In the field of catalysis, precise control over the properties of the catalyst is essential as any changes in the chemical composition, particle size, or surface area might drastically change its catalytic activity. Development of catalysts for electrochemical conversion of hydrogen, e.g., the hydrogen evolution reaction (HER) or the oxygen reduction reaction (ORR), is one of those research areas where reproducible catalysts are highly important as the dominating catalyst materials belong to the Platinum Group Metals (PGM) [3], [4]. The high cost of these catalysts inflicted by the price of PGMs makes it even more important to achieve the target properties as any catalytic performance that is not ideal will hinder further commercialization of hydrogen as a possible alternative to fossil fuels [3]. Reducing costs by optimizing the catalytic activity, getting reproducible nanoparticles, and scaling up the catalyst production are therefore paramount if PGMs are to be used in future electrochemical conversion devices for hydrogen [1].

The synthesis of catalysts for electrochemical conversion of hydrogen is predominantly based on the reduction of aqueous solutions of metal salts by a strong reducing agent such as sodium borohydride (NaBH_4_) [5], [6], [7], [8]. Other methods involve utilizing high temperatures to decompose the solvent into reducing species which will reduce the metal salts in a controlled manner. One of the most common examples of such a synthesis method is the polyol-synthesis [9]. However, to successfully achieve the desired properties, additives such as surfactants or stabilizers must be used [10]. This adds to the total cost of the catalyst by virtue of the price of the additive itself and the effort required to remove the additive post synthesis [10]. Also, the introduction of an additional component in the synthesis makes scaling the production more difficult as careful considerations must be taken into account to ensure reproducibility.

An alternative to the current nanomaterial synthesis methods is the sonochemical synthesis where high power ultrasound is used to reduce metal salts to metal nanoparticles [11], [12], [13], [14], [15]. Cavitation bubbles formed in the aqueous medium by ultrasound creates local environments of extreme temperatures and pressures upon collapse, resulting in the splitting of water molecules into hydrogen radicals (H·) and hydroxyl radicals (OH·). By applying a radical scavenger, like ethanol which consists of a polar part (OH-group) and a non-polar part (hydrocarbon chain), it will be situated at the bubble solution interface [16]. When the short-lived radicals are generated inside these bubbles they will be scavenged at the bubble solution interface and turn the scavenger into a reducing radical itself. The resulting radical is referred to as a secondary radical, and it will have a longer lifetime than primary radicals. The secondary radicals will therefore be able to diffuse into the solution, and operate as a continuously generated reducing agent for nanoparticle synthesis [16].

There are mainly two effects of ultrasound which can affect the nanoparticle synthesis; the sonochemical effects arising from radical formation during cavitation, and the mechanical effects caused by the shock wave pressure of the collapsing bubbles. The sonochemical effects are mostly influencing the rate of reduction, while the mechanical effects may affect the agglomeration process. The sonochemical effects of ultrasound have been studied quite extensively and several different groups report an optimum in the sonochemical effects between ultrasonic frequencies of 200 kHz and 500 kHz [14], [17], [18], [19]. At these optimum frequencies the particle size of sonochemically synthesized nanoparticles is expected to reach a minimum due to the increased reduction rate, which have been shown for gold nanoparticles [14] and ruthenium nanoparticles [17]. However, these works have used five frequencies spanning a wide range (20 kHz–1 MHz) rendering it difficult to pinpoint an exact optimum. Limiting the range of frequencies to the proposed optimal range for sonochemical radical formation would therefore be beneficial to determine an exact optimum frequency for sonochemical synthesis of nanomaterials. This is especially interesting for platinum (Pt) nanoparticles as its excellent properties as HER- and ORR-catalysts could really benefit from an optimized and robust synthesis method to justify the high cost of Pt. This can be achieved by conducting a thorough investigation of the frequency dependence of the properties of Pt-nanoparticles synthesized sonochemically. To the best of our knowledge, no such investigation has been performed for the sonochemical synthesis of Pt-nanoparticles. As for the mechanical effects, there are two phenomena which can affect the agglomeration process. First is the formation of high velocity microjets which will cause erosion or deagglomeration of the target material. However, microjet formation can only occur when the size of the target material is larger than the collapsing bubble (micro meter size) [20]. The next mechanical effect is the shock waves created by collapsing bubbles. Being multi directional, these are less violent than the microjets, but are also capable of deagglomeration. In addition these shock waves have also been found to aid agglomeration through the facilitation of interparticle collisions as was shown by Suslick et al. [20], [21]. The shock waves can therefore lead to both deagglomeration of larger agglomerates and facilitate agglomeration of primary particles. Agglomeration of primary particles on the nano scale is especially interesting in the field of catalysis where available surface area is key to achieving a higher catalytic activity [22]. As shock wave pressures are highly frequency dependent [23], assessing the effect of ultrasonic frequency on primary particle size and agglomeration could therefore provide valuable information on which ultrasonic frequencies to chose to achieve ideal catalyst properties, and which frequency range can be used for high reproducibility.

In this work we want to investigate if any frequency in the proposed optimum range (200 kHz–500 kHz) for radical formation can be used to synthesize highly reproducible Pt-nanocatalysts. This is achieved by assessing the combined contributions from the sonochemical effects and the mechanical effects of ultrasound. The combined effect of ultrasound on primary particle properties and agglomeration will therefore serve as a measure of how reproducible the sonochemical synthesis of Pt-nanoparticles is. The primary particle properties were evaluated based on measurements of the particle sizes through scanning/transmission electron microscopy (S(T) EM) and X-ray diffraction (XRD), and the catalytic activity towards the HER from electrochemical measurements. As for agglomeration, agglomerate sizes were obtained from dynamic light scattering (DLS), and agglomerate surface area from the electrochemical surface area (ECSA). S(T) EM micrographs were used to get an idea of the shape of the agglomerates.

## 2 Experimental

### 2.1 Sonochemical synthesis setup

**Fig. 1:**
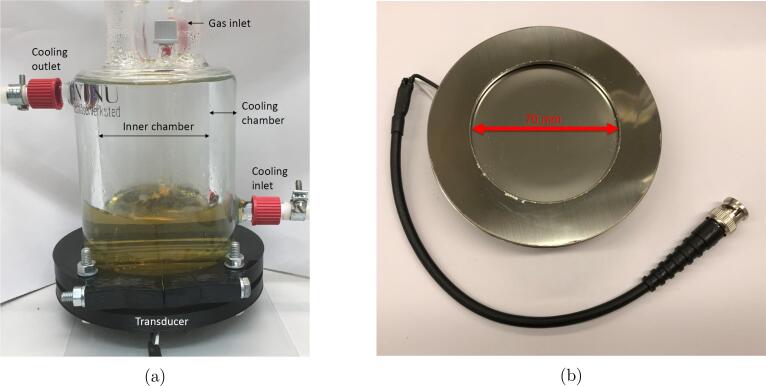
Ultrasound setup used to synthesize Pt-nanoparticles at different ultrasonic frequencies (a), along with one of the ultrasound transducers (b).

The experimental setup for the sonochemical synthesis is shown in Fig. 1. The frequency specific plate transducers (210 kHz, 326 kHz, 408 kHz, and 488 kHz) from Honda Electronics are made from a stainless-steel alloy (SUS304) which is highly corrosion resistant. These transducers were mounted on the bottom of a borosilicate glass reactor being in direct contact with the solution in the inner chamber of the reactor. The issue of eroded particles entering the system due to transducer erosion from micro jet formation has been shown to occur previously [24], but it was only observed at low ultrasonic frequencies (20 kHz) with high acoustic powers (43 W). The lower mechanical effects of ultrasound at higher frequencies (⩾210 kHz) and lower acoustic powers (11.8 W) are therefore not expected to cause erosion of the transducer. This was also confirmed from the absence of a constant baseline shift in the absorbance spectra (Fig. S1). In the outer chamber, water maintained at 20°C was circulated using a compact recirculating chiller from QSonica to maintain a constant solution temperature throughout the system.

### 2.2 Synthesis

Pt-catalysts were synthesized sonochemically using ultrasonic frequencies of 210 kHz, 326 kHz, 408 kHz, and 488 kHz. For each frequency, the acoustic power transmitted to the reactor was measured by calorimetry as described in Section 2.5. All plate transducers were operated at an acoustic power of 11.8 W (which would correspond to 0.307 W cm^−2^ or 0.059 W cm^−3^ for the geometry of our cell.) (Figure S2). The temperature during ultrasonication was maintained at 20°C throughout the entire experiment. For each synthesis 200 mL of 2 mmol dm^−3^ PtCl_4_ (99.9 % basis, Alfa Aesar) was prepared in a 0.8 mol dm^−3^ ethanol (96 %, GPR rectapur, VWR) solution where ethanol acts as a radical scavenger. Complete scavenging of the primary radicals by 0.8 mol dm^−3^ ethanol was ensured as shown by a separate dosimetry experiment as described in the supporting information (Figure S3). Prior to ultrasonication, the solution was purged with argon (Ar 5.0) gas for 10 min. Ar-gas was also supplied for the entire duration of the experiment. In order to achieve sufficient amounts of Pt-nanoparticles for further characterization the sonication time was set to 180 min. The Pt-nanoparticles were then extracted from solution by use of an Eppendorf 5810 R centrifuge operated at 12,000 rpm for 15 min. The particles were redispersed in ethanol and water and centrifuged an additional three times. The particles were then dried at room temperature before being ground into a fine powder using a mortar and pestle.

### 2.3 Physical characterization

The sonochemical synthesis of Pt-nanoparticles is expected to proceed from Pt(IV) to Pt(II) and then to Pt-nanoparticles. Primary radicals (OH· and H·) are scavenged by the scavenger (RH) and will act as reducing agents following the reactions below [25].

$$\mathrm{OH}\cdot (H\cdot )+\mathrm{RH}⟶H_{2}O(H_{2})+R\cdot$$

Pt(IV)+2R·⟶Pt(II)+2R

Pt(II)+2R·⟶Pt(0)+2R

The concentration of Pt(IV) and Pt(II) was therefore measured for all samples as a function of sonication time using UV–visible spectroscopy. A Thermo Scientific Evolution 220 UV–vis spectrophotometer was used to record the absorbance spectra. To distinguish the absorption peaks of Pt(IV) and Pt(II) a colorimetric technique was applied where an excess of potassium iodide (KI) (⩾99.0 %, ACS reagent, Sigma Aldrich) was added to the Pt(IV)/Pt(II) solution to form Pt iodide complexes with distinct absorption peaks in the visible spectrum [25]. The resulting PtI6^2−^ peak at 495 nm (∊ = 11,170 dm^3^ mol^−1^ cm^−1^) (Figure S9a) and the PtI4^2−^ peak at 388 nm (∊ = 4,600 dm^3^ mol^−1^ cm^−1^) [26] were related to the concentration of Pt(IV) and Pt(II), respectively through Beer–Lambert’s law.To avoid interference from the Pt(IV) peaks in the determination of the Pt(II) concentrations, the contributions of Pt(IV) was subtracted from the spectra. As a result of the uncertainties introduced with this step, the reported Pt(II) concentrations only serve to provide a qualitative assessment of the reduction mechanics of Pt, and is not considered quantitatively. Recording the concentrations of Pt(IV) as a function of sonication time also allowed for the determination of reaction rates at the different ultrasonic frequencies.

To examine the crystal structure, particle size, particle shape, and agglomerate size, XRD, S(T) EM, and DLS were utilized. For XRD and S(T) EM dilute dispersions of the different samples were drop cast onto a flat silicon wafer and a Cu formvar TEM grid from Ted Pella, respectively. The XRD measurements were performed with a Bruker D8 A25 DaVinci X-ray Diffractometer with CuKα radiation. A scan rate of 0.044°/step was used for 2θ-angles between 15°-75° with a 0.3° fixed slit for 60 min. From the diffractograms, the mean crystallite sizes, *L*, were estimated using the Scherrer equation [27]

(1) $$L=\frac{K\lambda }{\beta \cos\theta }$$

where *K* is the shape factor with a value of 1.333 for spherical particles [27], λ is the wavelength of the incident X-rays, β is the full width at half maximum for the given peak, and θ is the Bragg angle of the given peak.

For S(T) EM measurements a Hitachi High-Tech SU9000 was used in brightfield mode with an acceleration voltage of 30 kV and an emission current of 0.7μA. High angle annular dark field scanning transmission electron microscopy (HAADF-STEM) was also performed on the 210 kHz sample and the 326 kHz sample in order to compare the respective particles in more detail. For these HAADF-STEM measurements, a Jeol JEM ARM200F was used with an acceleration voltage of 200 kV. DLS was performed using a Beckmann Coulter N5 submicron particle size analyzer where samples were diluted to achieve a particle count of approximately 2 × $10^{6}$ cps [28]. Presence of interfering dust particles was minimized by filtering the dispersions through a 2.7μm particle filter while working in an ISO 7 cleanroom. Ten consecutive measurements were performed for each sample and using the obtained autocorrelation function, the hydrodynamic diameter of the particle agglomerates was extracted.

### 2.4 Electrochemical characterization

For electrochemical characterization of the Pt-nanoparticles, catalyst inks were prepared by mixing 10 mg of Pt-catalyst with 475μL DI-water (Milli-Q, 18.2MΩcm), 475μL isopropyl alcohol (IPA) (technical, VWR), and 50μL Nafion 117 (5 wt% in mixture of lower aliphatic alcohols and water, Sigma Aldrich). 10μL aliquots of the ink were spin coated onto a glassy carbon rotating disc electrode (RDE, Pine Instruments) (area  = 0.196cm^2^) at 200 rpm in order to obtain a homogeneous dispersion of the catalyst on the electrode with a Pt loading of 0.5 mg cm^−2^. The electrochemical measurements were performed in an Ar-saturated solution of 0.5 mol dm^−3^ H_2_SO_4_ (95–97%, VWR) with a reversible hydrogen electrode (RHE) as the reference electrode and graphite as the counter electrode. An Ivium-n-stat potensiostat was used to record all data. During the measurements, a rotation rate of 1,600 rpm was used for the RDE. Measures were also taken to cover the solution surface with Ar during the experiments to prevent air from entering the electrolyte. In order to activate the catalysts and obtain a stable voltammogram, the working electrode was cycled 20 times between 0.07 V vs RHE and 1.5 V vs RHE with a scan rate of 50 mV s^−1^ [29]. The voltammograms obtained during the final cycles were then used to determine the electrochemical surface area, ECSA, of the catalysts assuming a monolayer adsorption of hydrogen, with $Q_{a}$ = 220 μC cm^−2^ [30]

(2) $$\mathrm{ECSA}=\frac{Q_{H}}{220\mu {\mathrm{Ccm}}^{-2}}$$

Linear sweep voltammetry was performed between 0.1 and −0.1 V vs RHE using a scan rate of 1 mV s^−1^. Due to the high current densities obtained at low potentials, IR-compensation was applied with values chosen as 85% of the series resistance obtained from impedance measurements. The overpotential required to reach 10 mA cm^−2^ ($\eta_{10}$) was then recorded for each sample and compared with each other.

### 2.5 Dosimetry and calorimetry

Prior to the nanoparticle synthesis, the ultrasound reactor and the different ultrasound transducers were subjected to dosimetry experiments and calorimetry experiments in an effort to determine the radical yield and transmitted ultrasonic power. Two different dosimeters were employed; Potassium iodide (Weissler) dosimetry [19], [31], [32], [33], and titanyl sulfate dosimetry (TiOSO_4_) [34]. For Weissler dosimetry a 0.1mol dm^−3^ KI solution was oxidized to I3^−^ by OH-radicals formed during ultrasonication at the different ultrasonic frequencies [19]. I3^−^ having a strong yellow colour (∊ = 26,000 dm^3^ mol^−1^ cm^−1^) could therefore easily be quantified through UV–visible spectroscopy and Beer Lambert’s law. No molybdate catalyst was used for Weissler dosimetry to avoid the contribution from H_2_O_2_ as this is already accounted for by titanyl sulfate dosimetry. Instead Weissler dosimetry will indicate the amount of OH radicals that actually escape the cavitation bubbles.

As for titanyl sulfate dosimetry, only DI-water was subjected to ultrasonication. As the radicals being formed have no added species to react with, the radicals recombine themselves, and one of the dominating products is hydrogen peroxide (H_2_O_2_) due to the combination of two OH-radicals [34]. To detect the H_2_O_2_ being formed, an excess of TiOSO_4_ (1.9 %–2.1 %, Sigma Aldrich) was added to the sample after ultrasonication. The TiOSO_4_ reacts with H_2_O_2_ and forms a peroxotitanium (IV) complex with a strong absorption peak at 411 nm [34]. The molar absorption coefficient of this complex was measured in a separate calibration experiment with commercial H_2_O_2_ to be ∊ = 787dm^3^ mol^−1^ cm^−1^ (Figure S9b). Similarly to the Weissler method, samples were extracted at various time intervals and measured with UV–visible spectroscopy to determine the [OH·] ([OH·]= 2[H_2_O_2_]), and therefore the radical generation rate.

For the calorimetric measurements of the ultrasonic power, 200 mL of DI water was sonicated for 2 min at various amplitudes for all frequencies while measuring the temperature increase. Assuming all acoustic energy is transformed to heat the acoustic power can be calculated as [35]

(3) $$P_{\mathrm{acoustic}}={\mathrm{mC}}_{p}{\left(\frac{\mathrm{dT}}{\mathrm{dt}}\right)}_{t=0}$$

where (d*T*/d*t*)_t=0_ is the temperature slope of water per unit of sonication time (at *t* = 0) in K s^−1^; m is the mass of the water used in g and *C*_p_ is the specific heat capacity of water (4.186 J g^−1^ K^−1^). All measurements were repeated three times.

## 3 Results

**Fig. 2:**
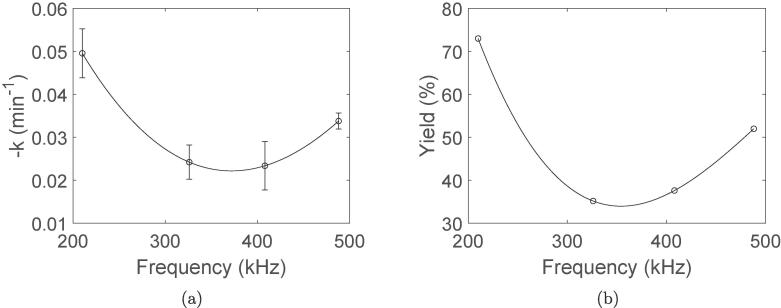
Rate constant (k) for the reduction of Pt(IV) (a), and the yield of Pt-nanoparticles (b) plotted against ultrasonic frequency. The error bars are equal to the respective standard deviations, and the lines through the datapoints are drawn with cubic spline interpolation to guide the eye.

The reduction rate constants (k) for the sonochemical reduction of Pt(IV) to Pt(II) obtained at different ultrasonic frequencies are shown in Fig. 2a. These values were extracted from the UV–visible absorbance spectra and the Pt(IV) concentration profiles provided in the supporting information (Figure S4 and Figure S5). Application of the lowest ultrasonic frequency (210 kHz) leads to significantly faster Pt reduction compared to the other frequencies. However, at the highest frequency (488 kHz) the reduction rate appears to increase again. The yield of the resulting Pt-nanoparticles are also plotted against applied ultrasonic frequency in Fig. 2b, and exhibit the same trend as the reduction rate constants.

**Fig. 3:**
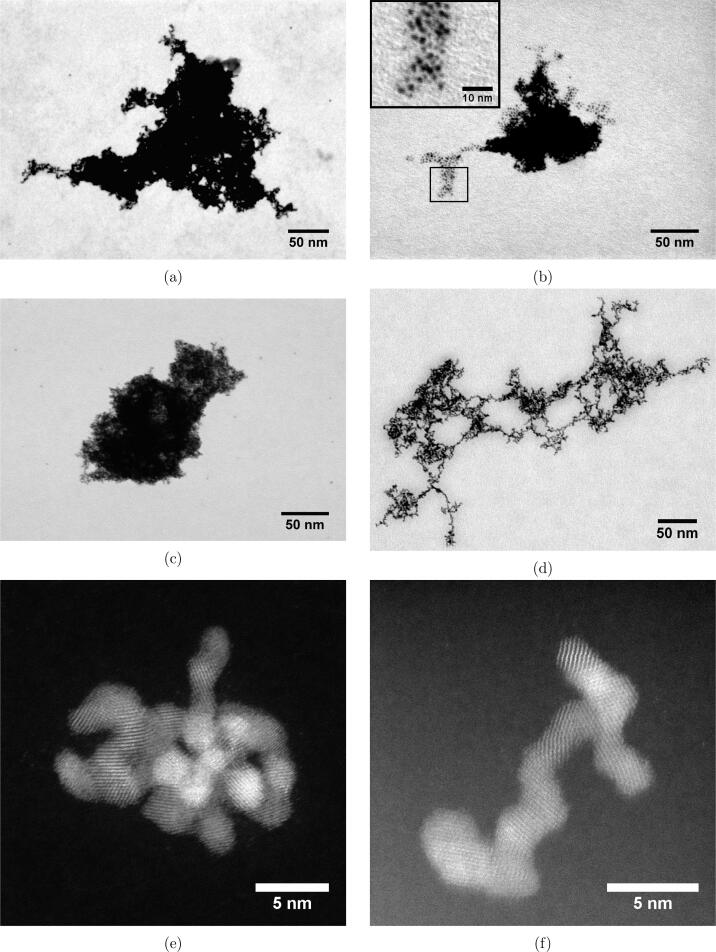
Bright field scanning electron microscopy micrographs of Pt-nanoparticles synthesized at 488 kHz (a), 408 kHz (b), 326 kHz (c), and 210 kHz (d). High angle annular dark field scanning transmission microscopy micrographs were also acquired for Pt-nanoparticles synthesized at 326 kHz (e), and 210 kHz (f). An inset of clearly resolved primary particles have been included for the 408 kHz sample (b).

S(T) EM micrographs of all Pt-samples are shown in Fig. 3a–d. From these micrographs we can see that the applied ultrasonic frequency during the synthesis does not affect the microstructure of the primary particles nor their size as they all exhibit a spherical shape with a size of (1.9 ± 0.3) nm. This is more apparent from the inset shown in Fig. 3b where the primary nanoparticles are more easily resolved compared to the bulky structure of the agglomerate. Even though there are no significant differences in the respective primary particles, the agglomerates formed at 210 kHz appear to be less dense than for the other frequencies. More examples are provided in the supporting information (Figure S6).

HAADF-STEM micrographs of Pt-nanoparticles synthesized using ultrasonic frequencies of 326 kHz and 210 kHz are shown in Fig. 3e and Fig. 3f, respectively. The way in which agglomeration occurs appear to be somewhat different for the two samples. For the 210 kHz sample, the agglomerates seem to form long strands, whereas for the 326 kHz sample the agglomerates become more clustered together as was also observed for the agglomerates in the S(T) EM images.

**Fig. 4:**
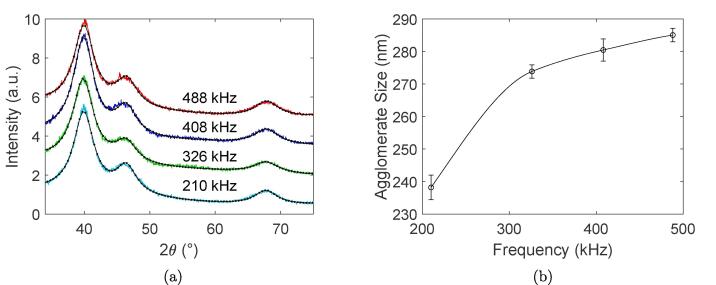
X-ray diffractograms of Pt-nanoparticles synthesized at ultrasonic frequencies of 488 kHz, 408 kHz, 326 kHz, and 210 kHz (a). Average agglomerate sizes of Pt-nanoparticles synthesized at ultrasonic frequencies of 488 kHz, 408 kHz, 326 kHz, and 210 kHz as obtained from dynamic light scattering (b). The line through the datapoints for dynamic light scattering is drawn with cubic spline interpolation to guide the eye.

The X-ray diffractograms of all Pt-samples are plotted together in Fig. 4a. These results show that all samples exhibit the same crystal structure of fcc Pt indicated by the peak positions at 40.0° (111), 46.5° (200), and 67.9° (220). Assuming a spherical shape of the crystallites as was observed from the S(T) EM micrographs, the subsequent analysis of the peaks using the Scherrer equation (Eq. 1) revealed that all samples displayed an average crystallite size of 2.1 nm which is very similar to the sizes obtained from S(T) EM. Results from dynamic light scattering highlighting the mean agglomerate sizes of the Pt-nanoparticles are shown in Fig. 4b. The figure clearly shows that the Pt-nanoparticles synthesized at the lowest ultrasonic frequency of 210 kHz exhibits a smaller agglomerate size compared to the other ultrasonic frequencies where the agglomerates appear to reach a constant value.

**Fig. 5:**
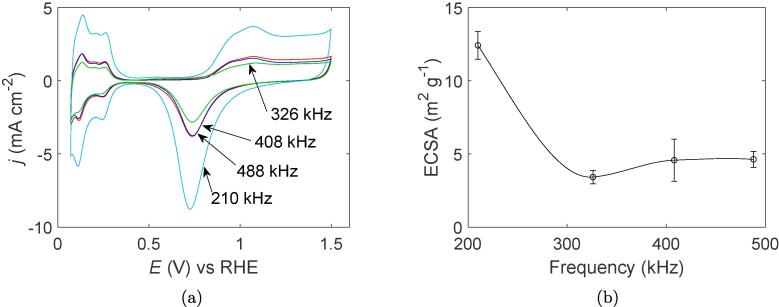
Cyclic voltammograms of Pt nanocatalysts synthesized at different ultrasonic frequencies (a), and the corresponding electrochemical surface area (b). The line through the datapoints is drawn with cubic spline interpolation to guide the eye.

The cyclic voltammograms (CV) normalized for the geometric surface area, and the corresponding electrochemical surface area for all samples are shown in Fig. 5a and Fig. 5b, respectively. Significantly higher current densities are observed for the 210 kHz sample compared to all the other samples which also leads to an ECSA nearly three times as high as the other samples. As for the other frequencies, the voltammograms are practically overlapping with each other which is also reflected in their similar electrochemical surface areas.

**Fig. 6:**
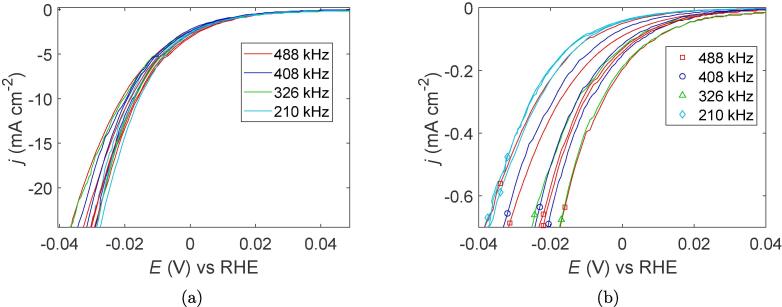
Linear sweep voltammograms of Pt nanocatalysts synthesized at different ultrasonic frequencies with current densities normalized for the geometric surface area (a), and electrochemical surface area (b).

Linear sweep voltammograms normalized for the geometric surface area and the ECSA for all samples are shown in Fig. 6a and Fig. 6b, respectively. No significant differences were observed between the four samples when the current densities were normalized for the geometric surface area as can be seen from the overlap of the curves and the similar overpotentials at 10mA cm^−2^ equal to (19 ± 2) mV. When normalizing the current densities for the ECSA, however, the performance of the 210 kHz catalyst towards hydrogen evolution was worse than the other frequencies. In addition, the 210 kHz catalyst displays a much higher degree of reproducibility compared to the other frequencies as indicated by the near overlap of the curves at 210 kHz. More variation is observed for the other frequencies, and no significant differences between the 326 kHz, 408 kHz, 488 kHz catalysts can be observed.

**Fig. 7:**
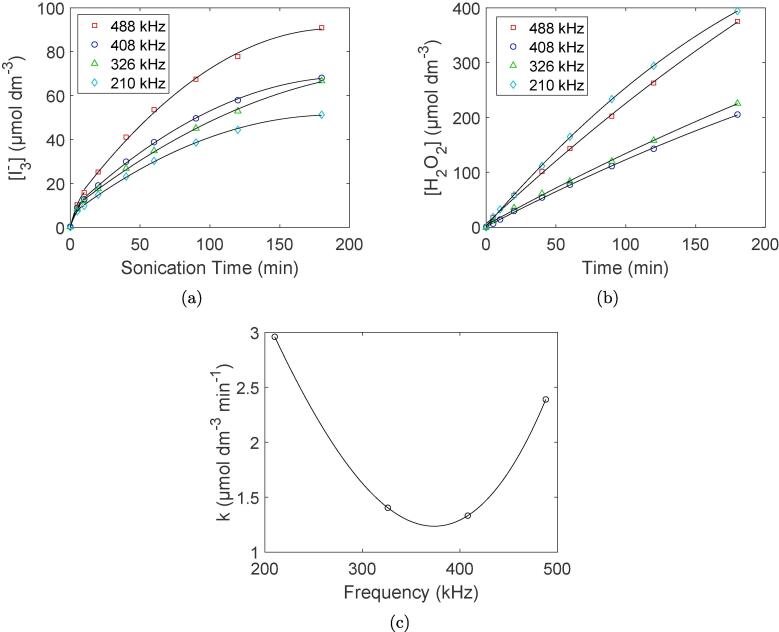
Concentration profiles of I3^−^ (a), the concentration profiles of H_2_O_2_ (b), and the reaction rate of OH· to H_2_O_2_ (c) for different ultrasonic frequencies. For the concentration profiles (a and b), fitted curves have been included for the respective datapoints. For the rate figure (c), the lines through the datapoints are drawn with cubic spline interpolation to guide the eye.

KI-dosimetry results for the different ultrasonic frequencies are shown in Fig. 7a. Concentration profiles were generated from UV–visible absorbance spectra provided in the supporting information (Figure S7). All concentration profiles deviate from linearity after a few minutes of sonication with values starting to flatten out towards the end of the 3 h sonication period. The final I3^−^ concentration for the different systems appear to be lowest at 210 kHz and highest at 488 kHz.

TiOSO_4_-dosimetry results for the different ultrasonic frequencies are shown in Fig. 7b-c, and are also extracted from UV–visible absorbance spectra provided in the supporting information (Figure S1). The concentration profiles are all approximately linear over the entire ultrasonication period with the exception of the lowest ultrasonic frequency (210 kHz), which flattens out slightly towards the end of the experiment. As for the rate of H_2_O_2_ formation, the fastest rate is observed at 210 kHz followed by 488 kHz. The 326 kHz and 408 kHz systems both display the slowest rate. This is in contrast to what is observed for KI-dosimetry where the 210 kHz system displayed the lowest I3^−^ concentration after 3 h.

For KI-dosimetry, I3^−^ is formed in situ and mostly by reacting with OH-radicals. Any H_2_O_2_ formed in the process from recombination of OH radicals will therefore not contribute to the I3^−^ concentration. The lower concentration of I3^−^ observed at 210 kHz may therefore be due to a faster recombination of OH radicals. However, as an interfacial radical scavenger (ethanol) was used in the actual synthesis, radicals are scavenged before they recombine as was also shown with a separate dosimetry experiment (Figure S3). KI-dosimetry is therefore suitable for the detection of OH-radicals when using solution based radical scavengers. The titanyl sulfate dosimeter therefore provides a more accurate estimation of the radical generation rate than KI-dosimetry for this particular synthesis.

## 4 Discussion

The agglomeration of Pt-nanoparticles appears to change from diffusion limited growth at lower ultrasonic frequencies to reaction limited growth at higher ultrasonic frequencies due to the frequency dependent mechanical effects of ultrasound. This transition follows from the clear difference in frequency dependence of the agglomerate size (Fig. 4b) from dynamic light scattering and the electrochemical surface area (Fig. 5b) from electrochemical measurements. Small porous agglomerates with large surface areas are characteristic of diffusion limited growth, while large compact agglomerates are characteristic of reaction limited growth. In addition, it was found from HAADF-STEM and S(T) EM micrographs (Fig. 3) that the lower ultrasonic frequency displays a more open, less compact agglomerate structure compared to the other frequencies.

From numerical simulations of agglomerate growth [p.29–31] [36] two distinct growth mechanisms can be found; diffusion limited growth and reaction limited growth. For diffusion limited growth, agglomerates will form upon contact between primary particles and therefore result in a more porous agglomerate, while for reaction limited growth the primary particles will have a lower probability for agglomeration and will therefore have time to diffuse into more energetically favourable surface sites. This creates a more compact structure.

Such a change in agglomeration can be attributed to the mechanical effects of ultrasound. Previous reports on the agglomeration formation from ultrasound suggests that the shock waves induced by ultrasound allows for high speed collisions of primary particles in such a way that they are fused together [20], [21]. Assuming laminar flow of the nanoparticles through the liquid, we can estimate the velocity, *v*, of the nanoparticles upon collision [21]

(4) $$v=\frac{\mathrm{rP}}{6\mu }\left(1-\exp\left(-\frac{9\mu \Delta t}{2\rho r^{2}}\right)\right)$$

where *P* is the shockwave pressure, *r* is the nanoparticle radius, μ is the viscosity of the liquid medium, ρ is the density of the nanoparticle, and Δt is the time it takes for the particle to collide with another particle. As the Pt-nanoparticle radius was found to be on the scale of 1 nm, the exponential term in Eq. 4 becomes negligible, and the collision velocity is given by

(5) $$v\approx \frac{\mathrm{rP}}{6\mu }$$

Using the shock wave pressures given by Merouani et al. [23] as an estimate of the pressures exerted on the nanoparticles during bubble collapse and the viscosity of water leads to collision velocities of 23.5m s^−1^ at 210 kHz, 12.8m s^−1^ at 326 kHz, 8.2m s^−1^ at 408 kHz, and 5.9m s^−1^ at 488 kHz. This is certainly not fast enough for the particles to melt together upon impact [21], but the kinetic energy supplied by the colliding particles could still aid agglomeration. Using the density of Pt (21.45g cm^−3^), and assuming spherical particles with a diameter of 1.9 nm, the kinetic energies attained for the different ultrasonic frequencies are estimated to be 0.13eV (12.5 kJ mol^−1^) at 210 kHz, 0.039eV (3.8 kJ mol^−1^) at 326 kHz, 0.016eV (1.5 kJ mol^−1^) at 408 kHz, and 0.008eV (0.8 kJ mol^−1^) at 488 kHz. As such, the lower ultrasonic frequency will provide the highest kinetic energy upon collision and therefore result in the highest probability for agglomeration. However, lower ultrasonic frequencies lead to fewer cavitation events per second due to the slower collapse of cavitation bubbles [23]. And this is only when we consider one cavitation event. As cavitation bubbles form due to the contraction and elongation of sound waves through the medium, the more contractions and elongations happening along the path of the sound wave the more cavitation bubbles will be generated [11]. A higher frequency (and shorter wavelength) will therefore cause more cavitation events.

In summation, the higher number of cavitation events at higher ultrasonic frequencies increases the supply of nanoparticles towards agglomeration, but due to the slower collision velocities, the probability of instantaneous agglomeration is lower compared to lower frequencies. As a result, more compact agglomerates, which is a characteristic of reaction limited growth, are formed at higher ultrasonic frequencies. Fewer cavitation events occur at lower frequencies resulting in less supply of nanoparticles to the agglomerate. However, the collision velocity is higher allowing agglomeration to occur at sites of lower energy, thus creating a more open agglomerate characteristic for diffusion limited growth. To strengthen this hypothesis molecular dynamics simulations might provide additional insight into particle collisions driven by the collapse of cavitation bubbles.

The impact of heat transfer in the region around the cavitation bubbles were also considered as a possible explanation for the differing agglomeration behaviour, but was not found to be significant. The idea is that metal nanoparticles in the hot spot region are subjected to high temperatures possibly melting and solidifying periodically depending on bubble collapse times (and ultrasonic frequency). However, due to the small size of the nanoparticles, any temperature gradients between the nanoparticles and the surrounding liquid will disappear completely on a much shorter time scale (picosecond scale) than the frequency dependent expansion times of the cavitation bubbles. This can be seen from Figure S8 in the supporting information which was calculated using the work of S. Paterson [37]. If agglomeration due to heat transfer were to be affected by the ultrasonic frequency, the heating and cooling rates of the nanoparticles would have to be at least on the same time scale as the duration of the cavitation events, which is not the case. We can therefore rule out heat transfer as an explanation to why the agglomeration behaviour is frequency dependent. The observed differences in agglomeration behaviour for different frequencies must therefore be due to purely mechanical effects.

For the same range of frequencies (210–488 kHz) that displayed a clear change in agglomeration behaviour, no significant differences were observed in the primary particle sizes of Pt-nanoparticles. All results related to the Pt primary particle size were found to be similar and therefore independent on the applied ultrasonic frequency within this range. This includes the crystallite sizes from XRD (2.1 nm) and the primary particle sizes from S(T) EM, (1.9 ± 0.3) nm. In addition, the overpotential required to reach 10mA cm^−2^ (geometric surface area) for the HER was the same for all samples (19 ± 2 mV) as can be seen from Fig. 6a. No apparent optimum ultrasonic frequency can therefore be identified within this frequency range. There is, however, a difference in the LSVs when the current is normalized for the ECSA (Fig. 6b), but this can be explained by the porosity of the agglomerates. El-Sayed et al. [38] showed that micro-bubbles can be trapped inside the pores of a catalyst effectively blocking the active sites. The porous agglomerates formed at 210 kHz would therefore be more prone to micro-bubble trapping during hydrogen evolution compared to the compact agglomerates formed at higher frequencies.

Even though previous reports [14], [17] have shown a frequency dependence on nanoparticle sizes, their frequencies span the entire sonochemical range from 20 kHz to 1 MHz providing very different synthesis conditions. Although the trends in radical formation rates (Fig. 7c) and reduction rates (Fig. 2a) for the different frequencies correspond well with these reports, the differences are apparently too small to be reflected in a detectable particle size change. An ideal frequency for producing the smallest Pt-nanoparticles may therefore exist in theory, but for all practical purposes, the entire frequency range between 210 kHz and 488 kHz can be used to obtain Pt-nanoparticles with the same primary particle size when using low acoustic powers (11.8 W).

Combining the sonochemical and mechanical effects of ultrasound therefore reveals that ultrasonic frequencies between 326 kHz and 488 kHz result in Pt-nanoparticles with near identical properties in terms of primary particle size and agglomeration behaviour. These frequencies therefore represent a range where high reproducibility can be expected. Going to even lower frequencies (210 kHz) revealed a change in the agglomeration process opening up the possibility of achieving more porous agglomerates, however the particle properties as well as the catalytic activity towards the HER remained the same. For applications where agglomeration is a problem, lower ultrasonic frequencies can therefore be used without changing the primary particle properties.

## 5 Conclusion

The frequency range used in this work clearly demonstrates a significant influence of the mechanical effects of ultrasound on the sonochemical synthesis of Pt-nanoparticles. At lower ultrasonic frequencies, the stronger mechanical effects are capable of changing the agglomeration mechanisms of the primary nanoparticles from reaction limited growth to diffusion limited growth resulting in smaller, more porous agglomerates. The lower ultrasonic frequency (210 kHz) also displayed a higher sonochemical effect as was proved by the higher Pt(IV) reduction rate, and higher radical formation rate. However, no significant changes in the primary nanoparticle size or shape could be found for any of the applied frequencies suggesting that the sonochemical synthesis yields highly reproducible Pt-nanoparticles over a very broad frequency range. This reproducibility was also reflected in the almost similar catalytic activity towards hydrogen evolution displayed by all samples. The sonochemical synthesis of monodisperse spherical Pt-nanocatalysts has therefore proven itself to be highly reliable over a broad frequency range (210 kHz–488 kHz) making it ideal for applications requiring a high degree of reproducibility.

## CRediT authorship contribution statement

**Henrik E. Hansen:** Conceptualization, Data curation, Formal analysis, Investigation, Methodology, Visualization, Writing – original draft, Writing – review & editing. **Frode Seland:** Supervision, Project administration, Validation, Writing – review & editing. **Svein Sunde:** Supervision, Resources, Writing – review & editing, Validation, Funding acquisition. **Odne S. Burheim:** Supervision, Validation, Writing – review & editing. **Bruno G. Pollet:** Supervision, Validation, Writing – review & editing.

## Declaration of Competing Interest

The authors declare that they have no known competing financial interests or personal relationships that could have appeared to influence the work reported in this paper.
