# Supplementary material for: Frequency controlled agglomeration of pt-nanoparticles in sonochemical synthesis
Source: Ultrason Sonochem. 2022 Mar 31;85:105991. doi: 10.1016/j.ultsonch.2022.105991 (PMC8980500; doi:10.1016/j.ultsonch.2022.105991)
Supplement: Supplementary data 1 [file mmc1.pdf]

Supporting Information

The absorbance spectra for $TiOSO_4$-dosimetry for all ultrasonic frequencies are shown in Figure S1. The peak absorbance at 411 nm is observed to increase over time in line with the expected increase of OH-radicals.

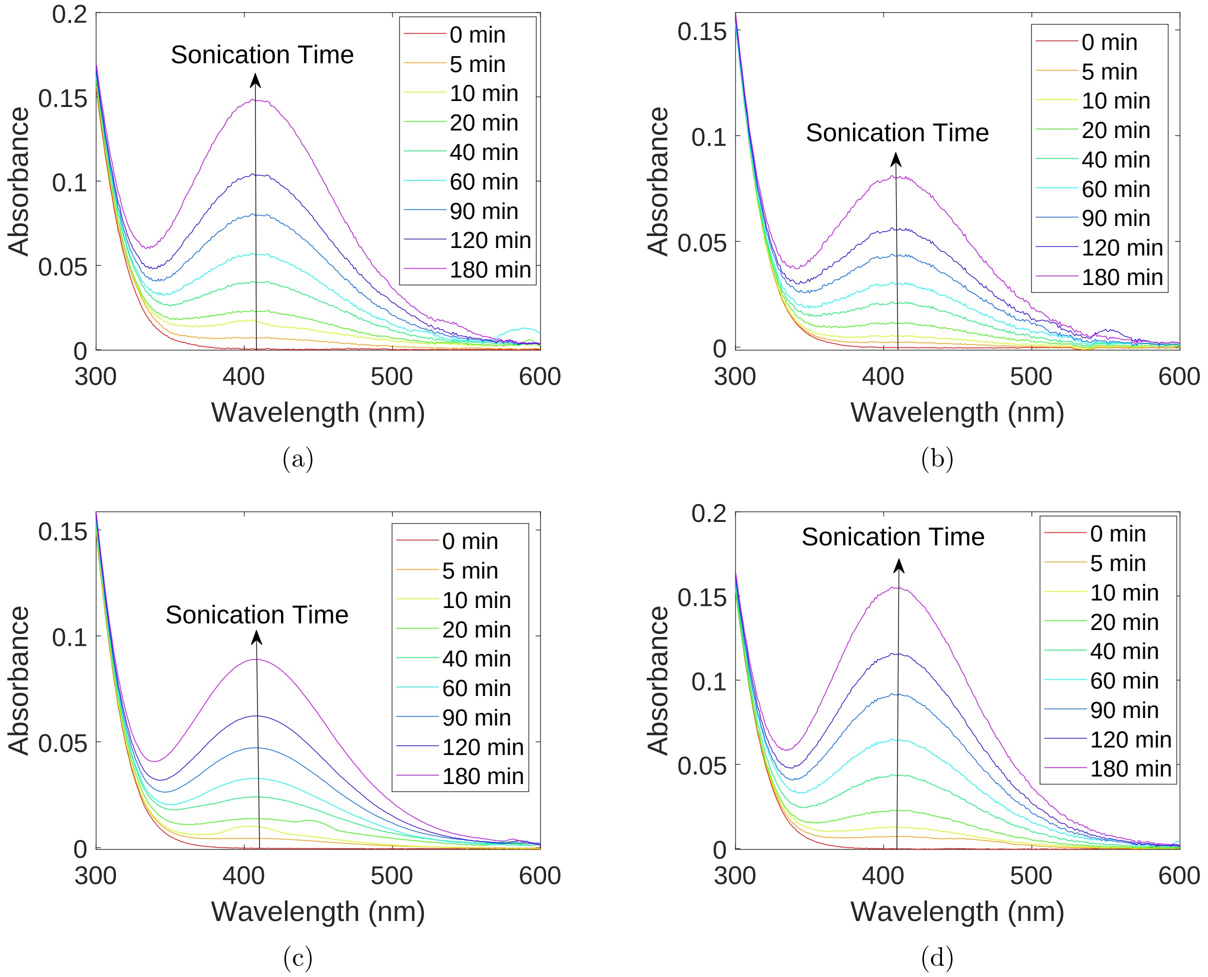

Figure 1: Absorbance spectra of a peroxotitanium (IV) complex plotted at different sonication times at frequencies of 488 kHz (a), 408 kHz (b), 326 kHz (c), and 210 kHz (d).

The ultrasonic power measurements, as calculated from Equation 3, are shown in Figure S2. In order to ensure sufficiently high powers for all frequencies, the maximum power (11.8 W) of the transducer with lowest maximum acoustic power (210 kHz) was chosen for all frequencies meaning amplitudes of 51%, 53%, 73%, and 100% were used at 488 kHz, 408 kHz, 326 kHz, and 210 kHz, respectively.

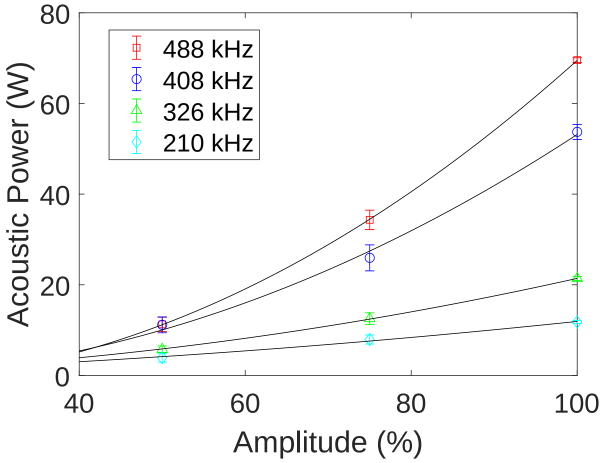

Figure 2: Calorimetric measurements of different ultrasonic frequencies as a function of amplitude.

To ensure that all primary radicals were scavenged by ethanol during the sonochemical synthesis, a separate dosimetry experiment was performed for the frequency which gave the highest radical generation rate (210 kHz) in which pure water was replaced by 0.8 mol dm^{-3} ethanol. The idea being that no H_2O_2 will be observed if ethanol manages to scavenge all primary radicals. This is reflected in the absence of a peak at 411 nm. In addition, the acoustic power was increased to 4 times the value used in the actual synthesis for good measure. The resulting absorbance spectra over a period of 3 hours are shown in Figure 3. The results confirm that no H_2O_2 is produced because all primary radicals are scavenged by ethanol.

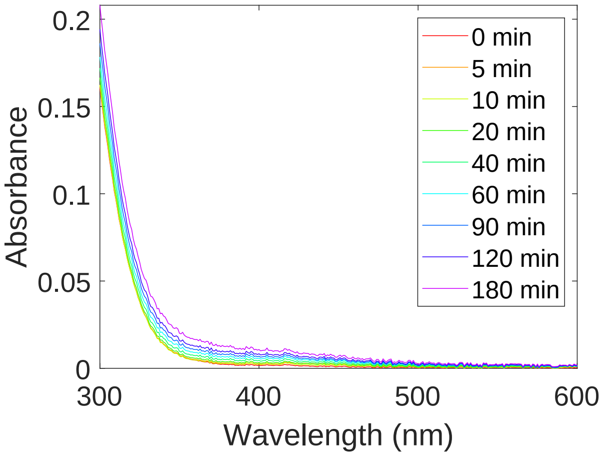

Figure 3: Absorbance spectra of a 0.8 mol dm^{-3} ethanol solution at different sonication times mixed with $TiOSO_4$.

Absorbance spectra of ${PtI_6}^{2-}$ and ${PtI_4}^{2-}$ acquired for all Pt-samples at different ultrasonication times (Figure S4). The peak at 495 nm corresponding to ${PtI_6}^{2-}$ and the emerging peak at 388 nm corresponding to ${PtI_4}^{2-}$ can be related to the concentration of Pt(IV) and Pt(II), respectively. These concentrations have been plotted against ultrasonication time for all Pt-samples in Figure S5. The

Pt(IV) concentration was found to decrease following an exponential decay. Simultaneously, the Pt(II) concentration was found to increase until it reached a maximum at intermediate ultrasonication times. The following decrease in the Pt(II) concentration is therefore a result of Pt-nanoparticle formation following the reduction of Pt(II) to Pt(0).

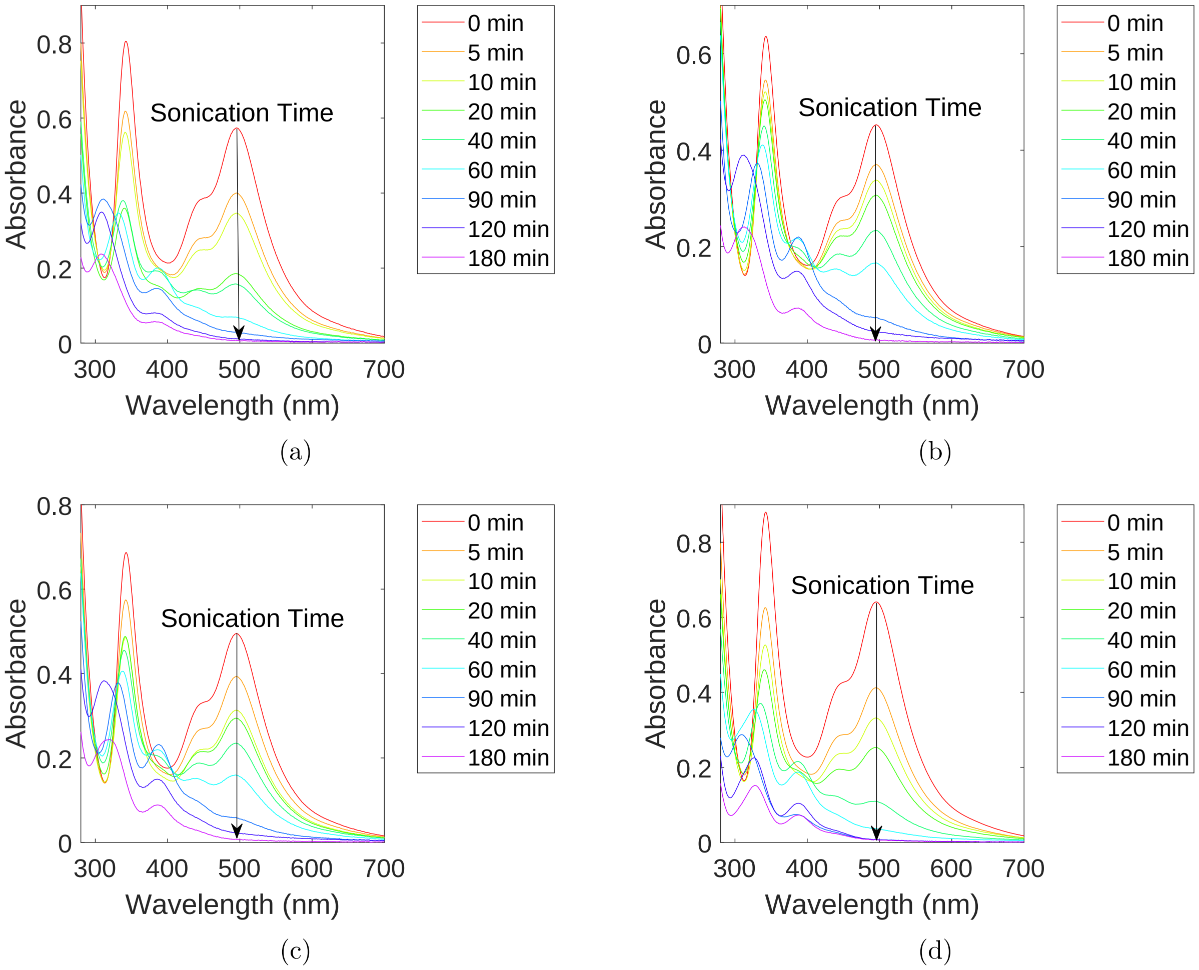

Figure 4: Absorbance spectra of $PtI_6{}^{2-}$ and $PtI_4{}^{2-}$ plotted at different sonication times at frequencies of 488 kHz (a), 408 kHz (b), 326 kHz (c), and 210 kHz (d).

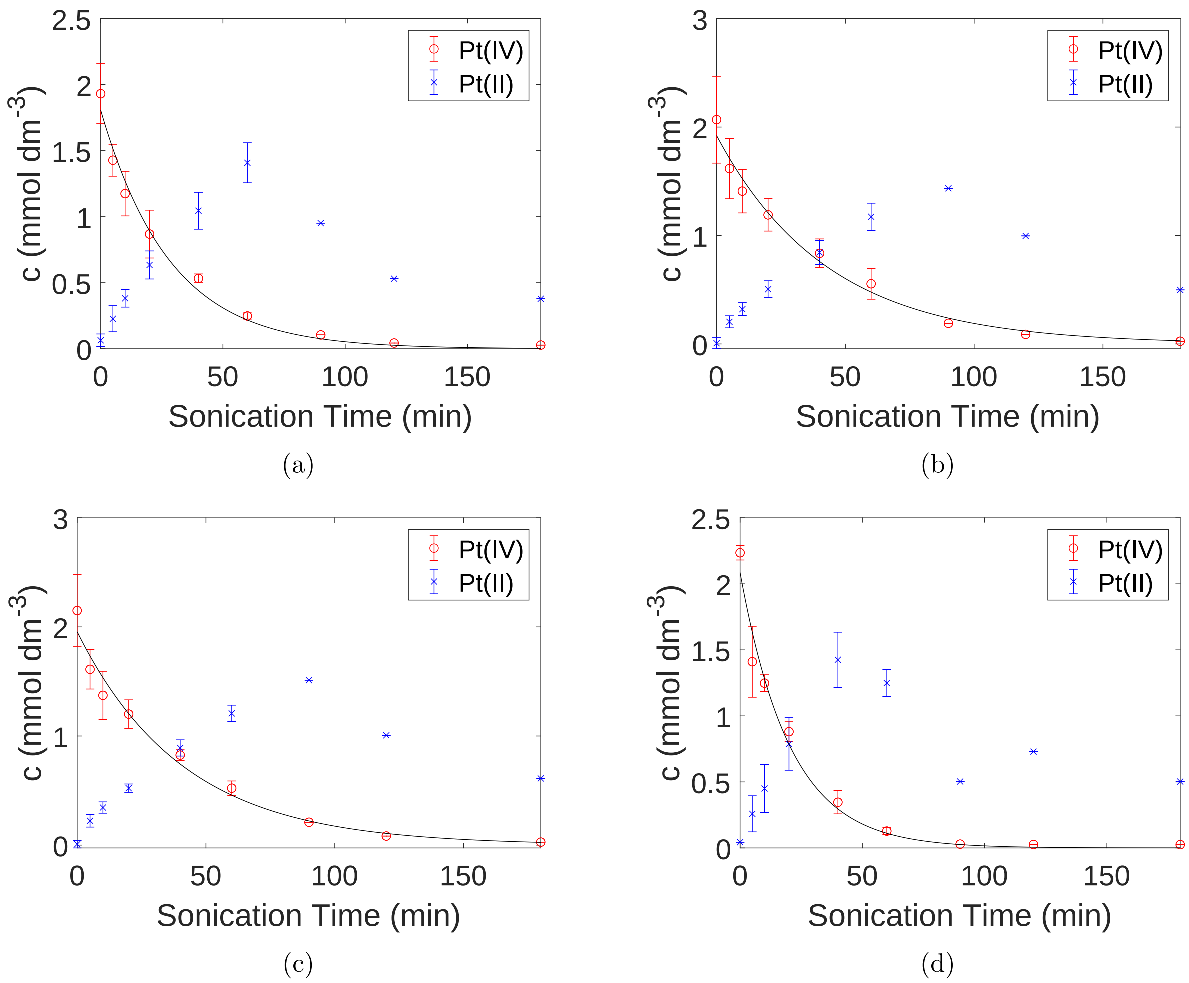

Figure 5: Concentration of Pt(IV) (o) and Pt(II) (x) plotted as a function of sonication time at frequencies of 488 kHz (a), 408 kHz (b), 326 kHz (c), and 210 kHz (d). The error bars are equal to the respective standard deviations.

Additional examples of S(T)EM micrographs of Pt-nanoparticles synthesized at different ultrasonic frequencies are shown in Figure S6.

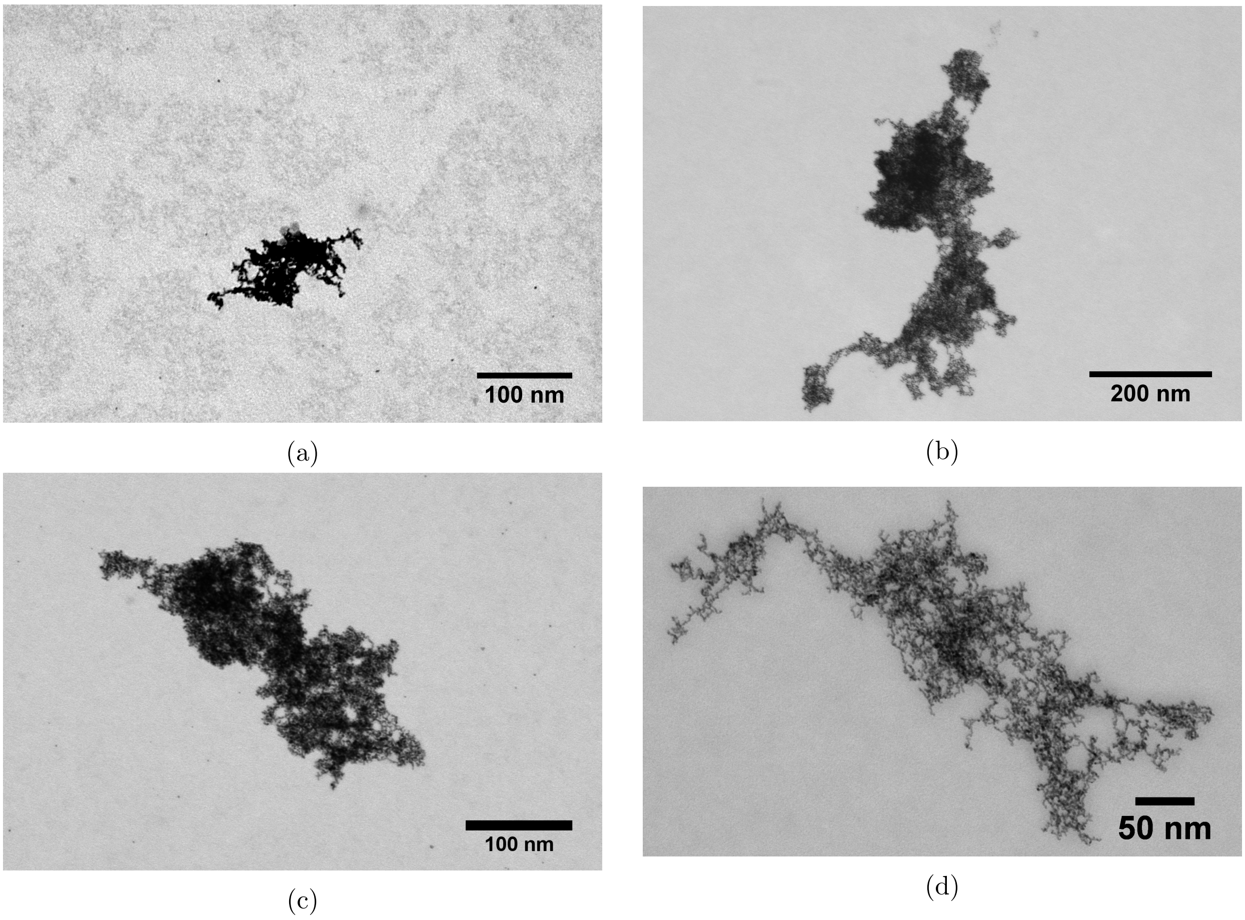

Figure 6: Bright field scanning electron microscopy micrographs of Pt-nanoparticles synthesized at 488 kHz (a), 408 kHz (b), 326 kHz (c), and 210 kHz (d).

The absorbance spectra for KI-dosimetry for all ultrasonic frequencies are shown in Figure S7. The peak absorbance at 350 nm is observed to increase over time in line with the expected increase of OH-radicals.

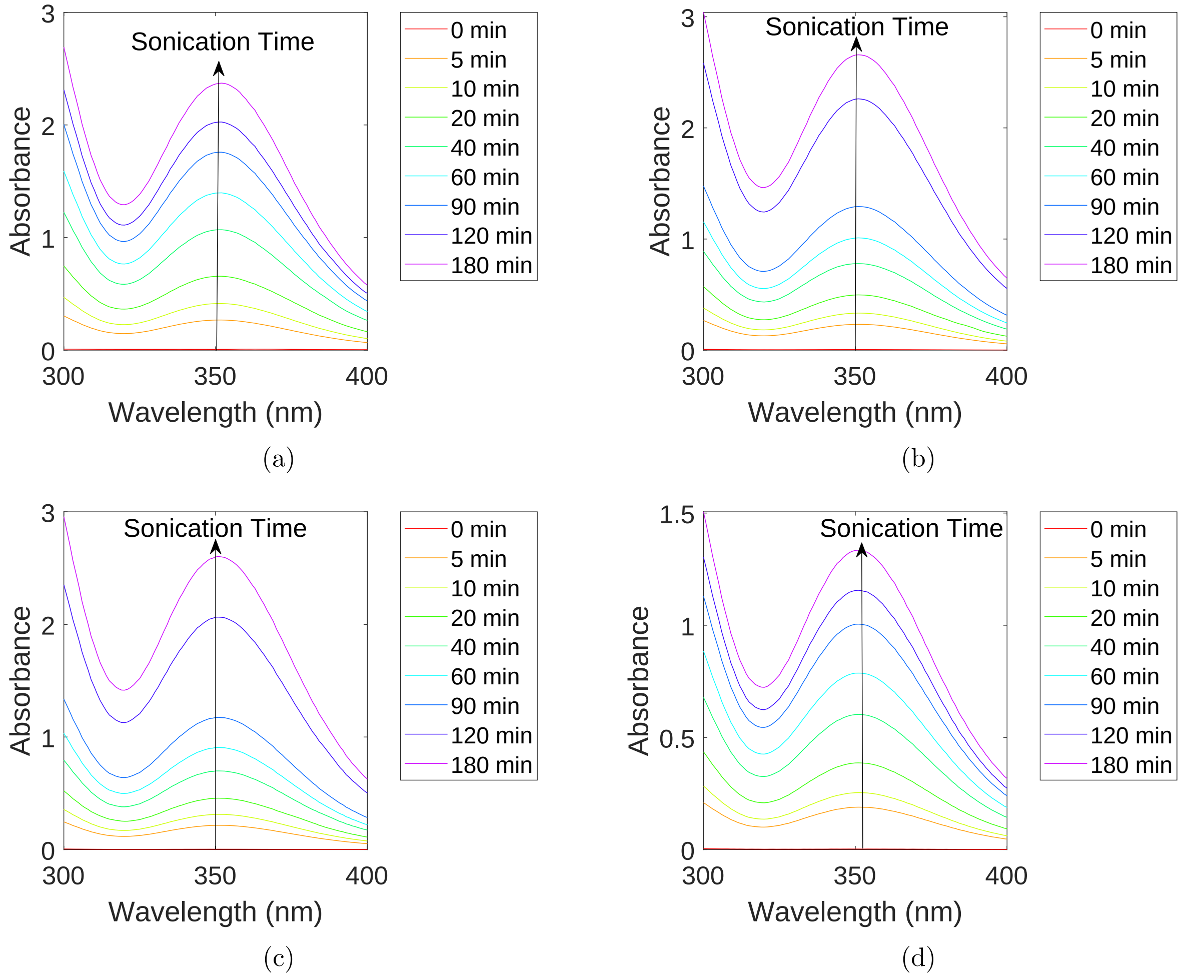

Figure 7: Absorbance spectra of $I_3{}^-$ plotted at different sonication times at frequencies of 488 kHz (a), 408 kHz (b), 326 kHz (c), and 210 kHz (d).

The temperature profiles from the center of a spherical Pt-nanoparticle to the surface for different cooling times are shown in Figure S8. The temperature of the surrounding liquid is set to 20 °C, and the profiles show a complete cooling of the particle within 1 ps. This estimation assumes that the Pt-nanoparticle is right next to the collapsing bubble, and experience the maximum bubble temperature of 5,000 K.

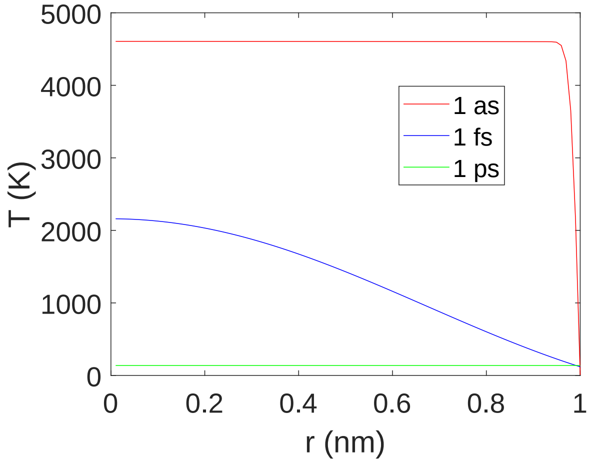

Figure 8: Temperature profiles of a Pt-nanoparticle after being heated by the temperatures generated by a collapsing cavitation bubble as a function of the particle radius. The center of the particle is at x = 0 nm, and the surface is at x = 1 nm. The temperature profiles are also shown for different cooling times (time after the heating was stopped).

Measurements of the molar extinction coefficient at 495 nm for ${PtI_6}^{2-}$ are shown in Figure S9a. From the slope of this curve, the molar extinction coefficient of ${PtI_6}^{2-}$ was determined to be ϵ = 11,170 $dm^3mol^{-1}cm^{-1}$. Measurements of the molar extinction coefficient at 411 nm for the $TiOSO_4$ complex formed due to interaction with H_2O_2 are shown in Figure S9b. From the slope of this curve, the molar extinction coefficient of this complex was determined to be ϵ = 787 $dm^3mol^{-1}cm^{-1}$.

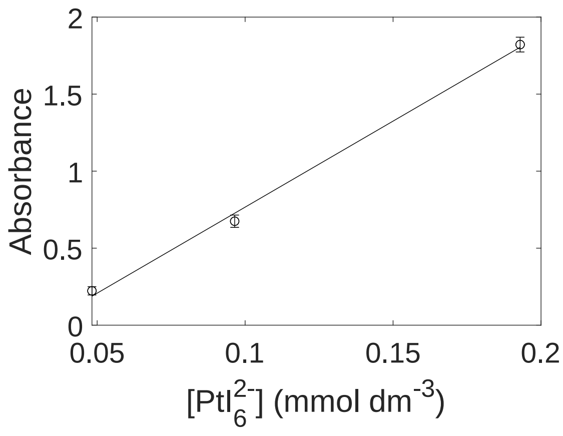

(a)

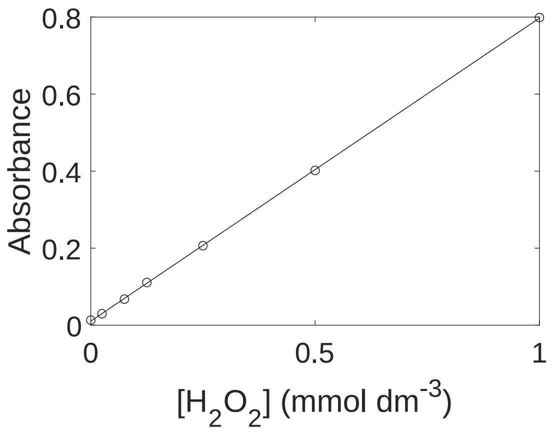

(b)

Figure 9: Absorbance of ${PtI_6}^{2-}$ at a wavelength of 495 nm measured for different known concentrations of Pt(IV) (a), and the absorbance of the $TiOSO_4$ complex at a wavelength of 411 nm measured for different known concentrations of H_2O_2.
